# Choline kinase inhibitors EB-3D and EB-3P interferes with lipid homeostasis in HepG2 cells

**DOI:** 10.1038/s41598-019-40885-z

**Published:** 2019-03-25

**Authors:** Alberto Sola-Leyva, Luisa C. López-Cara, Pablo Ríos-Marco, Antonio Ríos, Carmen Marco, María P. Carrasco-Jiménez

**Affiliations:** 1 Department of Biochemistry and Molecular Biology I, Faculty of Sciences, University of Granada, Av. Fuentenueva s/n, 18071 Granada, Spain; 2 Department of Pharmaceutical and Organic Chemistry, Faculty of Pharmacy, Campus of Cartuja, 18071 Granada, Spain; 3 Department of Cell Biology, Faculty of Sciences, University of Granada, Av. Fuentenueva s/n, 18071 Granada, Spain

## Abstract

A full understanding of the molecular mechanism of action of choline kinase α (ChoKα) inhibitors at the cell level is essential for developing therapeutic and preventive approaches for cancer. The aim of the present study was to evaluate the effects of the ChoKα inhibitors EB-3D and EB-3P on lipid metabolism in HepG2 cells. We used [methyl-^14^C]choline, [1,2-^1^^4^C]acetic acid and [2-^3^H]glycerol as exogenous precursors of the corresponding phospholipids and neutral lipids. [Methyl-^14^C]choline was also used to determine choline uptake. Protein levels were determined by Western blot. Ultrastructural alterations were investigated by transmission electron microscopy. In this work, we demonstrate that EB-3D and EB-3P interfere with phosphatidylcholine biosynthesis via both CDP-choline pathway and choline uptake by the cell. Moreover, the synthesis of both diacylglycerols and triacylglycerols was affected by cell exposure to both inhibitors. These effects were accompanied by a substantial decrease in cholesterol biosynthesis, as well as alterations in the expression of proteins related to cholesterol homeostasis. We also found that EB-3D and EB-3P lowered ChoKα protein levels. All these effects could be explained by the modulation of the AMP-activated protein kinase signalling pathway. We show that both inhibitors cause mitochondrial alteration and an endoplasmic reticulum stress response. EB-3D and EB-3P exert effects on ChoKα expression, AMPK activation, apoptosis, endoplasmic reticulum stress and lipid metabolism. Taken together, results show that EB-3D and EB-3P have potential anti-cancer activity through the deregulation of lipid metabolism.

## Introduction

Cancer is characterized by uncontrolled cell growth due to unrestrained proliferation and decreased apoptosis, as well as greater migration of cells capable of invading adjacent tissues and organs. That this disease is currently a leading cause of death worldwide, often due to chemotherapy resistance, highlights the urgency to seek new strategies and new drugs to fight the disease^1^. Each cancer is characterized by specific alterations that hamper developing a single strategy to combat them all. However, an alteration common to many types of cancer is an aberrant lipid metabolism. Thus, lipid metabolism may serve as a starting point for designing and developing new anticancer drugs^2,3^. In this way, in tumour cells and tumour progression, phospholipid biosynthesis is greater than in normal tissue^4,5^ and, more specifically, phosphocholine (PCho), and phosphatidylcholine (PC) levels rise in different cancers^6–8^. Furthermore, overexpression of the choline kinase α (ChoKα) isoform has been found in malignant cells and tumours of the liver, lung, colon, breast, prostate, and ovaries [Reviewed in^6^]. All such evidence makes the metabolism of choline and related compounds a metabolic hallmark associated with tumour onset and progression as well as the development of chemoresistance^9,10^. In this context, ChoKα has emerged as a marker for cancer progression and also as one of the most promising therapeutic target enzymes^9,11^.

ChoK participates in the biosynthesis of PC via CDP-choline, known as the Kennedy’s pathway^12^. First, choline enters the cells through several transporters^6,13^ and then can be phosphorylated to form PCho by ChoK activity. PCho is then activated to CDP-choline by CTP:phosphocholine cytidylyltransferase, and finally choline phosphotransferase catalyses the transfer of PCho to DAG to produce PC. In addition to participating in the biosynthesis of PC, ChoK also has other functions in regulating cell-signalling pathways. For example, it has been shown that downregulation of ChoKα attenuates the MAPK and PI3K/AKT signalling, which have been associated with cell proliferation^14^.

**Figure 1:**
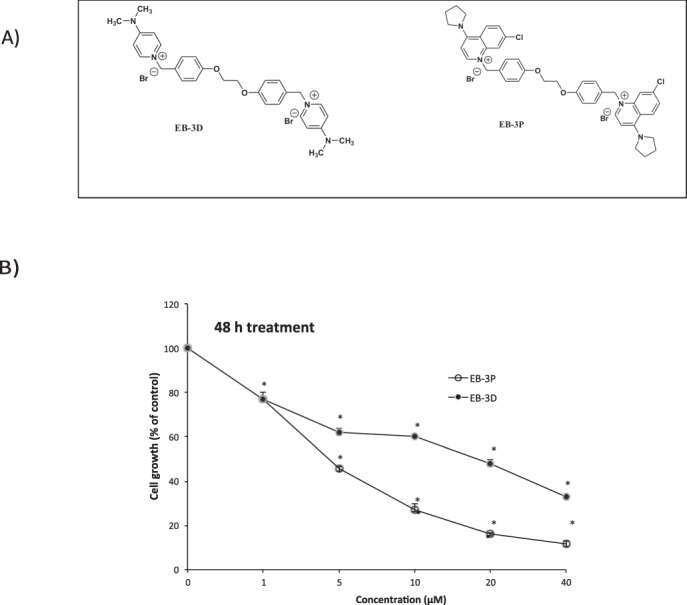
(**A**)Chemical structure of synthetic ChoKα inhibitors EB-3P and EB-3D. (**B**) Effects of EB-3D and EB-3P on HepG2 cell proliferation. HepG2 cells growing in the log phase were incubated with MEM/10% FBS in the presence or absence of different concentrations of ChoKα inhibitors for 48 h. Cell number was determined by crystal-violet staining and expressed as a percentage of the control cells. These experiments were performed twice in triplicate. ^*^P< 0.0001.

Given that ChoKα inhibition was considered to be of interest for inhibiting the growth and the invasive tumour phenotype, several laboratories began to synthesise compounds able to inhibit this enzyme. The first, hemicholinium-3, in addition to the inhibition of ChoKα activity blocks the sodium-dependent transport of choline and the synthesis of acetylcholine, with a high degree of side effects (Reviewed in^15^). Subsequently, bis-pyridinium (represented by MN58b) and bis-quinolinium (represented by RSM-932A) derivatives were synthesised, showing low or reduced toxicity in human tumours (Reviewed in^16^). Taking the MN58b and RSM-932A as patterns, and carrying out a detailed modelling study, we synthesised a series of new symmetrical biscationic compounds with the aim of increasing their polarity and solubility, improving inhibition of ChoKα enzyme, and consequently strengthening the antiproliferative effect on tumour-cell lines^17^. Among these, 1,1′-(((ethane-1,2-diylbis(oxy))bis(4,1-phenylene)), bis(methylene))-bispyridinium, or–bisquinolinium bromide, EB-3D, and EB-3P (Fig. 1A), respectively, as demonstrated in our laboratory, inhibited ChoKα activity in a low micromolar range^17^. Docking studies executed on both crystal structures, i.e. ChoKα 1/2 (PDB ID: 4BR3) and ChoKα 1/4 (PDB ID: 4CG8), showed that the two compounds could adopt a synclinal conformation of the linker, 1,2-dioxoethane fragment, which enabled it to be completely inserted into the enzyme.

Taking into account that ChoKα has become an attractive anticancer target, we analysed the cellular events triggered after treatment with the ChoKα inhibitors EB-3D and EB-3P and the underlying molecular mechanisms, paying attention to cell-death pathways closely related to PC and cholesterol metabolism. It is well known that compounds that affect phospholipid metabolism also alter cholesterol homeostasis, cholesterol metabolism being key in the decision-making process between cell proliferation and differentiation^18–20^. The study focuses on the HepG2 cell line, which retains many of the metabolic features of human hepatocytes and thus serves as an excellent model to study lipid metabolism^21^.

## Results

### EB-3D and EB-3P inhibit cell growth in HepG2 cells

The antiproliferative effect of EB-3D and EB-3P has been demonstrated in several cell lines^17^, but data are still unavailable for hepatic tumour cells. For an evaluation of the effect of these compounds on HepG2 cell proliferation, cells were incubated in the absence or presence of the inhibitors at concentrations of up to 40 μM for 48 h. The GI_50_ (half-maximal growth inhibitory concentration) value derived from the growth-inhibition curve of HepG2 cells were of 14.55 ± 2.13 and 4.81 ± 1.07 μM for EB-3D and EB-3P, respectively (Fig. 1B). This antiproliferative action could not be attributed to lysis as measured by LDH release into the medium (data not shown). The results demonstrate that in this cell line, the bisquinolinium derivative EB-3P had higher antiproliferative activity than did the bispyridinium compound EB-3D.

### Interference of EB-3D and EB-3P in phosphatidylcholine metabolism

**Figure 2:**
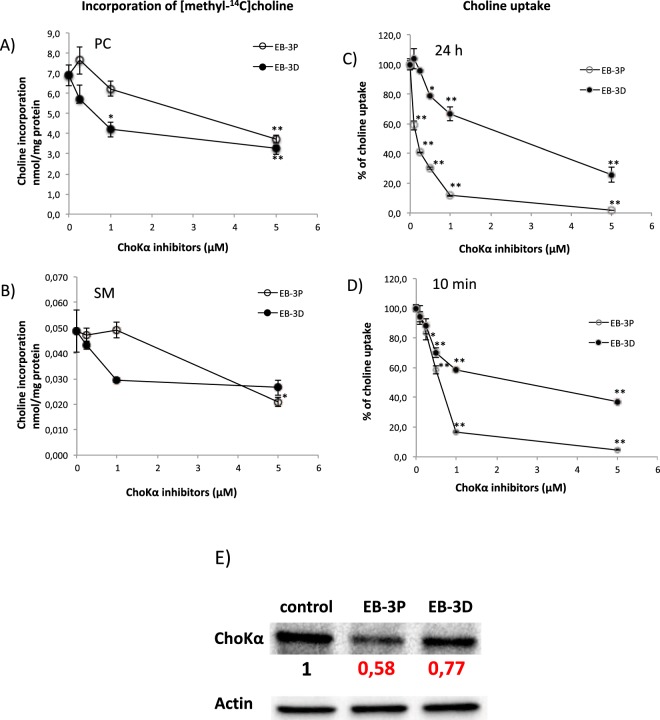
Effects of EB-3D and EB-3P on [methyl-^14^C]choline incorporation into phospholipids, choline uptake and ChoKα protein levels in HepG2 cells. Log-phase HepG2 cells were incubated with ChoKα inhibitors as described in Methods section. The incorporation of choline into PC (**A**) and SM (**B**) is expressed as nmol of choline incorporated per mg of cell protein. Choline uptake was determined in cells treated for 24 h (**C**) or 10 min (**D**) and expressed as a percentage of the control cells. Results represent the mean ± SEM of three independent experiments conducted in triplicate. ^*^P < 0.05, ^**^P < 0.001 when compared with control values. In E) it is represented the protein levels of ChoKα normalized to their respective β-actin level and expressed as x-fold change compared with the corresponding control ratio (1.0). The Western blot image shows a representative experiment repeated three times.

We examined the effect of EB-3D and EB-3P on the biosynthesis of PC by using choline as exogenous precursor. As expected, both EB-3D and EB-3P clearly inhibited the incorporation of radiolabelled choline into the final product of the biosynthetic pathway, the PC. Thus, as reflected in Fig. 2A, exposure of HepG2 cells to both synthetic compounds resulted in a dose-dependent reduction of choline incorporation into PC. The effects of EB-3P and EB-3D on choline incorporation were similar, with IC_50_ values of approximately 5 μM. Since transfer of PCho from PC to ceramide produces sphingomyelin (SM), the labelling of SM was also determined under the same conditions. Figure 2B shows that both EB-3P and EB-3D depress SM synthesis, although this effect proved significant only at amounts higher than 1 μM of the drugs.

### Inhibition of choline uptake by EB-3D and EB-3P

We also analysed the choline uptake in the HepG2 cell line. According to our results, in cells treated for 24 h with the inhibitors, choline uptake was strongly inhibited, registering IC_50_ values of 0.14 ± 0.01 μM for EB-3P and 1.21 ± 0.14 μM for EB-3D (Fig. 2C). Also, inhibition of choline uptake was detectable after 10 min of incubation with the inhibitors, as reflected in Fig. 2D. In any case, the action of EB-3P inhibitor is clearly stronger than that of EB-3D.

### Exposure to EB-3D and EB-3P affects the expression of ChoKα

Figure 2E represents images of ChoKα expression levels from HepG2 cells. To investigate the effect of EB-3D and EB-3P on ChoKα protein levels, we treated cells with inhibitors, and cell lysates were prepared as described in Methods section. Treatment of HepG2 cells with EB-3D or EB-3P resulted in a 23% or 42% lowering of ChoKα protein levels, respectively.

In addition, we determined the effect that EB-3D and EB-3P exert on the PC levels. As might be expected, the inhibitors significantly lowered the PC levels in HepG2 cells (Table 1). EB-3P reduced the amount of PC by 15 and 31% at 10 and 30 μM, respectively, whereas EB-3D resulted in a minor inhibition (c. 14% at 10 and 30 μM).

**Table 1:** Effects of EB-3D and ED-3P on PC levels. Log-phase HepG2 cells were incubated with ChoKα inhibitors for 48 h. Levels of PC were determined as described in methods section.

		nmol PC/mg of protein
Control		154,43 ± 2,79
EB-3P	10 μM	128,47 ± 2,05^a^
	30 μM	106,18 ± 4,12^b^
EB-3D	10 μM	133,63 ± 2,65^b^
	30 μM	131,66 ± 7,60^a^

The results are expressed as nmol of PC per mg of cell protein and represent the mean ± SEM of two independent experiments conducted in triplicate. ^a^P < 0.05 ^b^P < 0.005 when compared with control values.

### Interference of EB-3D and EB-3P in phospholipid and neutral lipid metabolism

**Figure 3:**
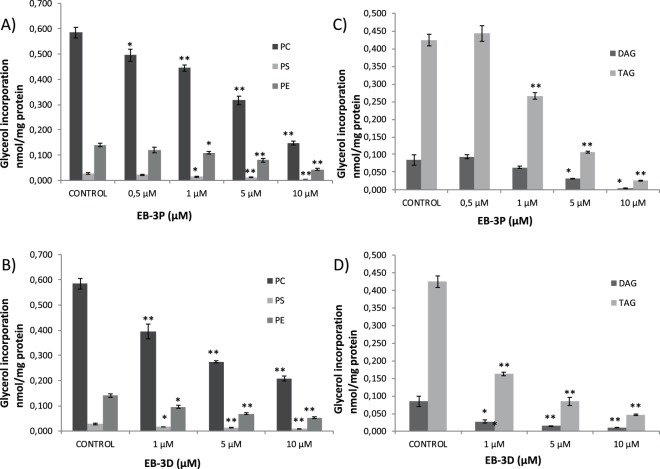
Effects of EB-3D and EB-3P on [2-^3^H]glycerol incorporation into lipids in HepG2 cells. Log-phase HepG2 cells were exposed to ChoKα inhibitors for 24 h. Then, cells were incubated with [2-^3^H]glycerol as described in Methods section. The incorporation of glycerol into phospholipids (**A**,**B**) and neutral lipids (**C**,**D**) is expressed as nmol of glycerol incorporated per mg of cell protein and represent the mean ± SEM of two independent experiments conducted in triplicate. ^*^P < 0.05 ^**^P < 0.005 when compared with control values.

We used radiolabelled glycerol as an exogenous precursor to explore the effects of EB-3D and EB-3P on both phospholipid and neutral lipid biosynthesis. Both inhibitors markedly reduced the incorporation of glycerol into PC and phosphatidylethanolamine (PE) (Fig. 3). Thus, it is not surprising that the biosynthesis of phosphatidylserine (PS), a phospholipid synthesised via a base-exchange reaction that converts pre-existing phospholipids (i.e. PC and PE) into PS (Reviewed in^22^) was clearly reduced in treated cells (Fig. 3A,B). Notably, the biosynthesis of the neutral lipids triacylglycerol (TAG) and DAG also was markedly affected by both EB-3P and EB-3D in a dose-dependent manner. In this case, however, EB-3D appeared to be more strongly inhibitor, registering IC_50_ values of less than 1 μM, which are clearly lower than those of EB-3P in HepG2-treated cells (Fig. 3C,D).

### Inhibition of cholesterol biosynthesis by EB-3D and EB-3P

**Figure 4:**
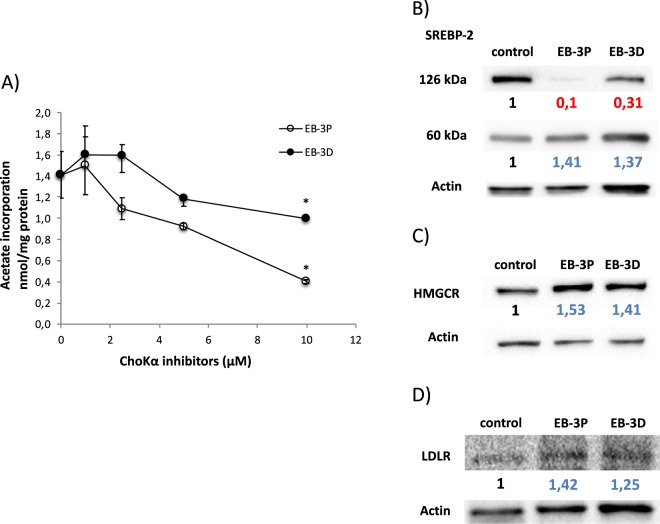
Effects of EB-3D and EB-3P on [1,2-^14^C]acetic acid incorporation into cholesterol and SREBP-2, HMGCR and LDLR protein levels in HepG2 cells. Log-phase HepG2 cells were incubated with ChoKα inhibitors for 24 h. Then, cells were incubated with [1,2-^14^C]acetic acid as described in the Methods section. The incorporation of acetate into cholesterol (**A**) is expressed as nmol of acetate incorporated per mg of cell protein and represent the mean ± SEM of two independent experiments conducted in triplicate. ^*^P < 0.05 when compared with control values. In (**B**) SREBP-2, (**C**) HMGCR and (**D**) LDLR are represented the protein levels normalized to their respective β-actin level and expressed as x-fold change compared with the corresponding control ratio (1.0). Western blots show a representative experiment repeated three times.

To determine whether these inhibitors altered cholesterol metabolism, we measured cholesterol biosynthesis by the incorporation of radiolabelled acetate into cholesterol. Notably, both inhibitors significantly affected the incorporation of acetate into cholesterol, showing a decrease in cholesterogenic activity. However, EB-3P was more potent (70% inhibition at 10 μM concentration), than EB-3D, showing 29% inhibition at the same concentration (Fig. 4A).

### Exposure to EB-3D and EB-3P affects the expression of proteins related to cholesterol homeostasis

These results led us to analyse the levels of the main proteins involved in cholesterol homeostasis, such as sterol regulatory element-binding protein 2 (SREBP-2), 3-hydroxy-3-methylglutaryl-coenzyme A reductase (HMGCR) and LDL receptor (LDLR). Cholesterogenesis is known to be transiently induced by the translocation of the SREBP-2 transcription factor from the endoplasmic reticulum (ER) (126 kDa precursor form) to the nucleus (60 kDa mature form)^23^. High expression levels of the mature SREBP-2 were detected after treatment with ChoKα inhibitors when compared with basal levels in untreated cells, whereas the membrane-bound precursor form decreased in parallel with the appearance of the released mature form (Fig. 4B). This increase in the active form of SREBP-2 was accompanied by an increase in the expression of its targets, HMGCR and LDLR (Fig. 4C,D).

### Alterations of autophagy-related proteins by EB-3D and EB-3P

**Figure 5:**
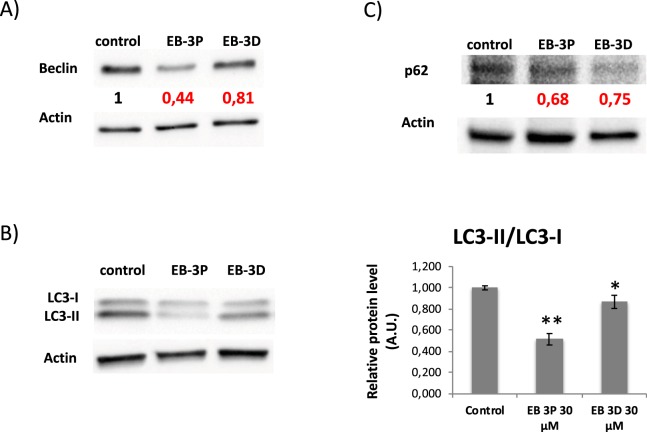
Effects of EB-3D and EB-3P on (**A**) Beclin, (**B**) LC3 and (**C**) p62 protein levels. HepG2 cells were incubated without any additions (control) or ChoKα inhibitors for 24 h. Protein levels in the samples were normalized to their respective β-actin level and expressed as x-fold change compared with the corresponding control ratio (1.0). Western blots show a representative experiment repeated three times. Ratios between the intensity of the bands corresponding to LC3-II and LC3-I are shown (right panel). ^*^P < 0.05, ^**^P < 0.001 compared to control value.

Autophagy is an intracellular process involved in the reutilizing of different cell components through the lysosomal pathway, a catabolic process that maintains cell homeostasis. In the present work we investigated the status of several autophagy-related factors, Beclin-1, microtubule-associated protein 1 light chain 3 (LC3), and p62, these being proteins that reflect the nucleation, elongation, and degradation stages of autophagy, respectively^24^. In our experiments, both inhibitors lowered Beclin-1 levels significantly at 24 h of exposure (Fig. 5A). Moreover, the conversion of LC3-I to LC3-II, the lipidated membrane-bound form, appears to be hindered by the inhibitors since the LC3-II/LC3-I ratio significantly decreased after HepG2 cells were exposed to inhibitors (Fig. 5B). In line with the above changes, the levels of p62 were significantly lower after the exposure of HepG2 cells to both EB-3D and EB-3P (Fig. 5C).

### Effects of inhibitors on apoptosis

**Figure 6:**
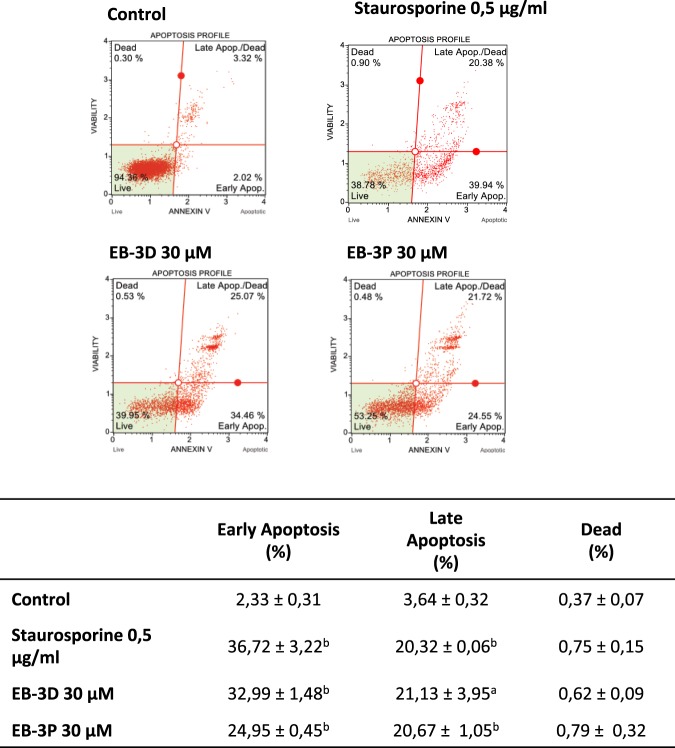
Effect of EB-3D and EB-3P on apoptosis in HepG2 cells. HepG2 cells were incubated in the absence and presence of 30 μM ChoKα inhibitors or 0.5 μg·mL^−1^ staurosporine for 24 h. Cells were collected and analysed by flow-cytometry to determine apoptosis, as described in the Methods section. Figure shows representative flow-cytometry images of the control and treated cells. The percentages of each cell population are included in a table in which results are expressed as the mean ± SEM of three independent experiments conducted in triplicate. ^a^P < 0.05, ^b^P < 0.005 when compared with control values.

We quantified the percentage of apoptosis in HepG2 cell cultures at basal levels and in the presence of the ChoKα inhibitors EB-3D and EB-3P. The apoptotic degree of HepG2 cells after treatment with ChoKα inhibitors is shown in Fig. 6. Exposure to the inhibitory compounds revealed a higher percentage of early and late apoptosis. As can be seen, percentage of cells in apoptosis at basal levels was low due to the normal cell death in culture, which did not exceed 4%.

### Effects of inhibitors on endoplasmic reticulum stress

**Figure 7:**
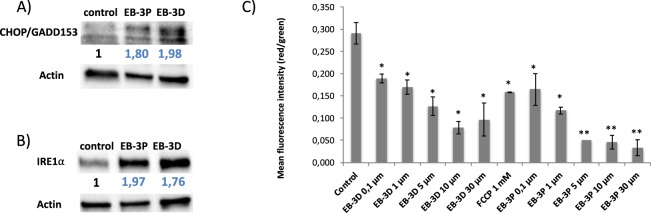
Effects of EB-3D and EB-3P on the expression of endoplasmic reticulum stress markers and on mitochondrial membrane potential in HepG2 cells. HepG2 cells were incubated without any additions or ChoKα inhibitors for 24 h. Protein levels of CHOP/GADD153 (**A**) and IRE1α (**B**) in the samples were normalized to their respective β-actin level and expressed as x-fold change compared with the corresponding control ratio (1.0). Western blots show a representative experiment repeated three times. For mitochondrial membrane potential determination we used JC-10 as described in Methods section. The ratio of red/green fluorescence intensity was used to determine the values of MMP. Results are expressed as the mean ± SEM of three independent experiments conducted in triplicate. ^*^P < 0.05, ^**^P < 0.001 when compared with control values.

To evaluate the effects of EB-3D and EB-3P on ER stress-signalling pathway, we determined levels of some proteins related to this process. HepG2 cells exposed to the drugs showed that CHOP/GADD153 (CCAAT/enhancer binding protein homologous transcription factor) and IRE1α (Inositol-requiring enzyme 1) protein levels significantly rose, as showed in Fig. 7A,B, respectively.

### Effects of inhibitors on the mitochondrial membrane potential and intracellular reactive oxygen species production

Next, we analysed the effects of EB-3D and EB-3P on mitochondrial membrane potential (MMP) and oxidative stress in HepG2 cells. The results revealed that neither of the two inhibitors influenced ROS production (data not shown). However, MMP was significantly diminished, with EB-3P again being more potent (Fig. 7C). Carbonyl cyanide-4-(trifluoromethoxy) phenylhydrazone (FCCP) was used as a positive control for MMP.

### Ultrastructural alterations produced by EB-3D and EB-3P

**Figure 8:**
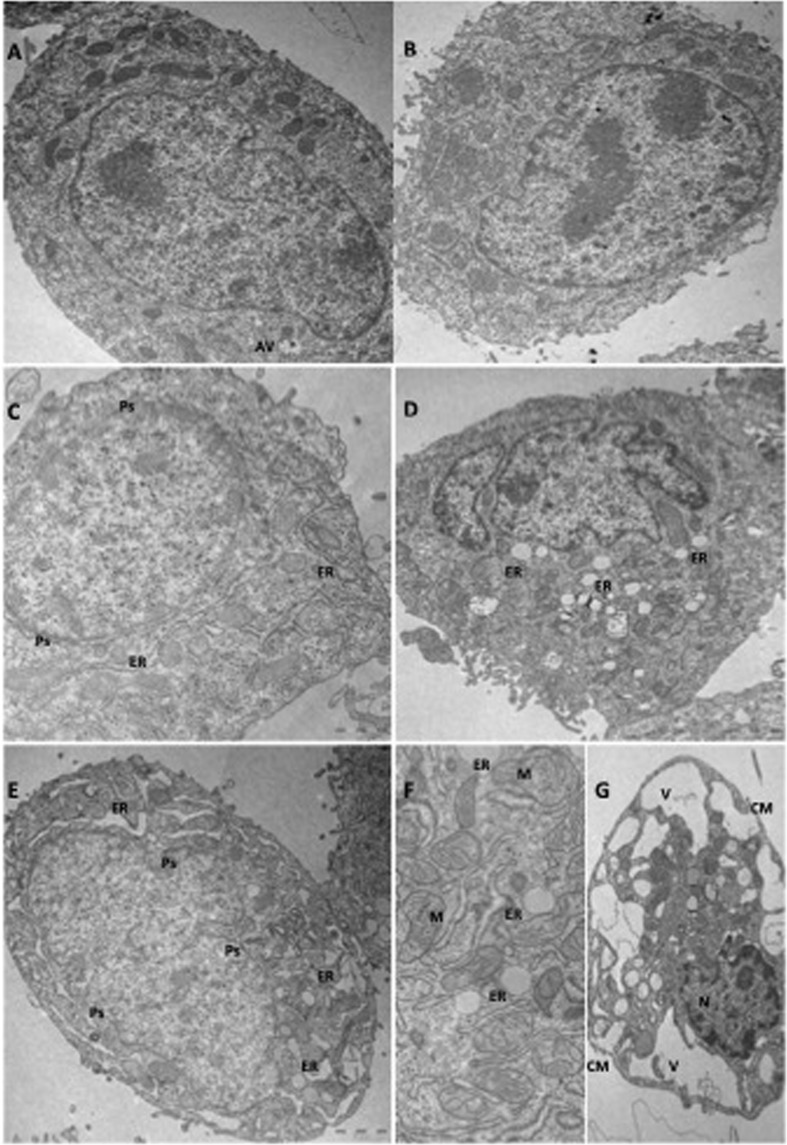
Ultrastructural alterations produced by ChoKα inhibitors. (**A**) Control HepG2 cells. (AV) Autophagic vacuole. (**B**) HepG2 cell exposed to EB-3D 10µM show ultrastructure similar to untreated cells. (**C**) Treatment with EB-3D 30 µM. Some dilatation of endoplasmic reticulum (ER) as well as of perinuclear space (Ps) is visible. (**D**) 24 h of exposure to EB-3P 10 µM. Cell shows a clear dilatation of ER cisternae and nuclear lobulation. (**E**) Treatment with EB-3P 30 µM increases the dilatation of ER. Ps is also dilated. (**F**) Detail of an EB-3P-treated cell showing wide ER cisternae and mitochondrial (M) rarefaction. (**G**) Cell in apoptotic stage induced by treatment with EB-3P. Picnotic nucleus (N), with many chromatinic bodies and perinuclear space notably dilated, is shown. Mitochondria are disorganised and great cytoplasmic vacuoles (V) appear as result of cell disorganization. Cell membrane (CM) rupture can be seen at some points.

To analyse the possible morphological changes caused by EB-3D and EB-3P exposure, we used transmission electron microscopy (TEM) in control and HepG2-treated cells with 10 µM and 30 µM of both ChoKα inhibitors for 24 h. The results are shown in Fig. 8. In A) the untreated control cell shows mitochondria having a dense matrix, many cisternae of rough ER, and a small autophagic vacuole (AV) at the bottom of the micrography. In B) the morphology was not significantly affected by exposure to EB-3D 10 µM since the cells showed an appearance similar to that of controls. In C) the cells treated with EB-3D 30 µM underwent some dilatation of the ER cisternae and had a less dense mitochondrial matrix. The perinuclear space also showed a dilatation with an increasing separation between the two nuclear membranes. Exposure to EB-3P resulted in a more notable effect on the ultrastructure of HepG2 cells. Thus, in D) the treatment of the cells with 10 µM of this compound prompted notable dilatation of the ER, and the loss of mitochondrial density was also patent. The cell nucleus appeared lobulated with dense chromatinic bodies adhering to the inner nuclear membrane. In E), the treatment at the highest concentration (30 µM) increased the dilatation of ER cisternae, the rarefaction of mitochondria, and the enlargement of the perinuclear space. F) shows a detail indicating the dilatation of mitochondria with disorganised cristae, a less dense matrix and the ER cisternae with a notable dilatation of inner space, reflecting ER stress. G) shows HepG2 cells at the apoptotic stage induced by treatment with EB-3P. A picnotic nucleus is visible with many chromatinic bodies adhering to the inner membrane and a notably dilated perinuclear space. The mitochondria look disorganized and great cytoplasmic vacuoles appear, as a result of cell disorganization. The cell membrane shows ruptures at some points.

### EB-3D and EB-3P modulate the AMP-activated protein kinase signalling pathway

**Figure 9:**
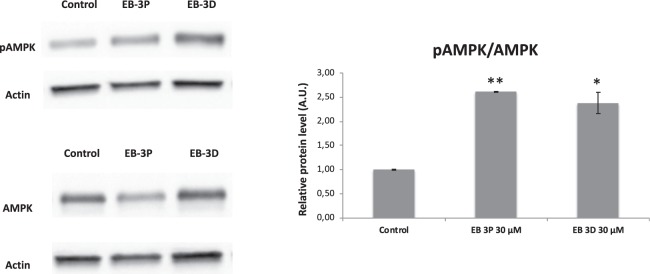
Effects of EB-3D and EB-3P on AMPK protein levels. HepG2 cells were incubated without any additions or ChoKα inhibitors for 24 h. The bands corresponding to pAMPK and total AMPK were scanned, and arbitrary units were assigned by densitometric analysis. The figure shows a representative experiment repeated three times (left panel). Ratios between the intensity of the bands corresponding to pAMPK and AMPK are shown and expressed as x-fold change compared with the corresponding control ratio (1.0) (right panel). ^*^P < 0.05, ^**^P < 0.001 compared to control value.

AMP-activated protein kinase (AMPK) is an intracellular energy-sensing kinase that is activated by AMP binding and phosphorylation of a threonine residue (Thr-172). To determine whether EB-3D and EB-3P were capable of modulate AMPK, we measured by Western blot the phosphorylation level of AMPK on HepG2 cells after 24 h of ChoKα inhibitor treatment. The results indicate that both compounds significantly raised pAMPK levels and consequently the pAMPK/AMPK ratio (Fig. 9). In addition, we determined the effect that EB-3D and EB-3P exert on the ATP levels. The inhibitors significantly lowered the ATP levels in HepG2 cells. EB-3P reduced the amount of ATP by 63 and 96% at 10 and 30 μM, respectively, whereas EB-3D resulted in a minor inhibition (c. 19% at 10 and 61% at 30 μM).

## Discussion

Lipids, required to maintain cell structure and provide energy, are also involved in cell signalling. Their metabolism generates a variety of biological intermediates that can regulate cell growth, proliferation, differentiation, survival, apoptosis, inflammation, and membrane homeostasis^25,26^. Disturbed lipid metabolism can cause diverse diseases, including a variety of cancers^2^.

Many tumours have been described to have an accelerated choline metabolism, the main consequence of which is a rise in the amount of choline-containing compounds^6,8,15^. In the last decade, with ChoKα considered an attractive target for novel anticancer therapies, a broad class of inhibitors of the isoenzyme ChoKα has been synthesised. Previously, we have reported that the bispyridinium and bisquinolinium derivatives of 1,1′-(((ethane-1,2-diylbis(oxy))bis(4,1-phenylene))bis(methylene)) containing a pair of oxygen atoms in the linker of their structure, EB-3D and EB-3P, respectively, inhibited ChoKα activity with a similar value of IC_50_ (1.00 ± 0.01 and 0.92 ± 0.01 μM, respectively), also exerting anti-neoplastic effects over an array of tumour-cell lines^17^. In the present work, we firstly evaluated the dose-dependent antiproliferative activity of these compounds against the HepG2 cell line. We selected this hepatoblastoma cell line for being an excellent model to study lipid metabolism^21^ and showing high mRNA-expression levels of ChoKα^27^. Our results indicate that HepG2 cells are less sensitive to EB-3D or EB-3P than are some other human tumour cells^17^, demonstrating that the extent of the cytotoxic effects of these agents depends upon the tumour-cell line.

The data compiled in the present work show that ChoKα inhibition by EB-3D or EB-3P depressed PC biosynthesis through the CDP-choline pathway, this also leading to a marked inhibition of SM production. These results agree with the lower levels of PC detected in our study after treatment with inhibitors. However, since choline is essential for PC biosynthesis, the intracellular choline availability could also limit the synthesis of this phospholipid. Notably, as shown, choline uptake was markedly inhibited in the presence of both ChoKα inhibitors not only after 24 h of treatment but also after only 10 min of exposure of HepG2 cells. All together, our results support the conclusion that EB-3D and EB-3P inhibit both the ChoKα and the choline transport activity, and that the dual action of these compounds on both processes is associated with a lower level of PC and consequently diminished cell proliferation.

Several works demonstrate that ChoKα downregulation with siRNA significantly diminishes cell viability, suggesting that the ChoKα protein levels are in part responsible for altering cell viability^9,10^. In this sense, Mori *et al*.^28^ have published data on a selective ChoKα inhibitor, V-11-0711, which significantly lowered PCho levels but did not reduce cell viability as long as ChoKα protein and PC levels were not lowered. Notably, in our study EB-3D and EB-3P decreased ChoKα protein and lowered PC levels.

Glycerol is a precursor of phosphatidate, a common intermediate in the synthesis of phospholipids and TAG. The use of glycerol as an exogenous substrate enables us to analyse the activity of both biosynthetic pathways simultaneously. As might be expected, EB-3D and EB-3P caused a significant decrease in the quantity of glycerol incorporated into phospholipids, as described in the Results section. This action was accompanied by a marked decrease in the incorporation of glycerol into TAG and DAG. This lower availability of DAG could in part contribute to the decline in phospholipid synthesis observed in our study.

The importance of the PC and cholesterol synthesis pathways in tumour proliferation is evident from previous studies conducted by our research group^18,20^. No data are available concerning the effects of ChoKα inhibitors on the biosynthesis of cholesterol, and thus the results presented in this paper demonstrate for the first time that EB-3D and EB-3P decrease the de novo synthesis of cholesterol. However, of the two inhibitors tested, EB-3P exerted stronger effects on cholesterol synthesis. In any case, the simultaneous reduction in the cholesterogenesis and synthesis of choline-containing phospholipids found in this study agree with reports by several researchers indicating that the concentration of cholesterol and phospholipids containing choline are regulated in a coordinated manner, since their relative proportions are essential in maintaining cell-membrane integrity^19^.

All these metabolic changes led us to investigate the status of AMPK, a cell-energy sensor activated in response to a variety of conditions that deplete cell energy^29^. This protein can also act as a metabolic tumour suppressor inhibiting cell growth in a wide variety of cancers^30,31^. In the present work, both EB-3D and EB-3P increases the phosphorylation and activation of AMPK in HepG2 cells. Actually, in our study, we found that both ChoKα inhibitors cause a concentration-dependent decrease in MMP not accompanied by an increase in ROS. In this way, as shown by TEM, mitochondria of HepG2-treated cells were clearly affected, showing disorganization and loss of density at the higher amount of inhibitors. The reduction of the MMP would depress ATP production and raise the AMP/ATP ratio. This implies a decrease in cell-energy reserves and thus a possible additional cause for the inhibition of PC biosynthesis, mainly because ChoKα activity is quite sensitive to changes in ATP concentration^32^. Moreover, the AMPK activation stimulates the pathways that generate ATP, while biosynthetic processes such as cholesterol and TAG biosynthesis are inhibited, as observed in our study. In relation to cholesterol metabolism, it has been widely documented that activated AMPK phosphorylates and inactivates the limiting enzyme of cholesterol biosynthesis, HMGCR^29^. These data agree with the inhibition of cholesterol synthesis found in our study after ChoKα inhibitor treatments. Cholesterol biosynthesis is also transcriptionally regulated by SREBP-2. In the absence of cholesterol the mature SREBP-2 migrates to the nucleus and acts as a transcription factor to promote the expression of target genes involved in cholesterol biosynthesis and uptake, such as HMGCR and LDLR^33^. Data from our study indicate that the inhibition of HMGCR by AMPK could lead to an initial reduction of intracellular cholesterol, activating SREBP-2 and increasing protein expression of its targets, HMGCR and LDLR, as occurs after statin treatment^34^. Moreover, our data agree with those of other authors^35,36^, who hold that AMPK activation leads to a compensatory increase in SREBP-2, and consequently in the transcripts of its target genes.

In addition to the importance of mitochondria in energy metabolism, they have also been recently shown to participate in regulating cell death. An alteration in MMP leads to the opening of pores in the mitochondria and the release of cytochrome c to the cytosol, which activates caspase-9. On being activated, caspase-9 in turn activates caspase-3, which triggers the last stages of apoptosis. The results from TEM and flow cytometry analyses reveal that HepG2 cells treated with inhibitors show an apoptotic process. However, we found that apoptosis induced by EB-3D or EB-3P cannot be attributed to a mechanism involving mitochondria-mediated pathways, since we did not detect caspase-3 activation (data not shown), suggesting caspase-independent cell death. It should be taken into account that apoptosis has also been linked to ER stress induction, as reported elsewhere^37^. It is well established that ChoKα inhibition in cancer cells induces ER stress and triggers apoptosis^37–39^. CHOP has been established as an acute ER stress marker^40^. In addition, IRE1α is ER stress sensor and is essential for the unfolded protein response and sufficient to trigger the apoptotic response^41^. Ultrastructural examination of HepG2 cells treated with both inhibitors revealed the well-characterised distortion of ER with cisternae that presents a notable dilatation of the inner space associated with ER stress. The results suggest that the drugs promote intensified ER stress response, sustained by CHOP and IRE1α production.

We have also investigated the possible involvement of another pathway of cell death, i.e. the autophagy on the EB-3D and EB-3P biological actions. As mentioned above, the fall in the levels of the autophagy-related proteins measured in our study clearly indicates that both EB-3D and EB-3P act as inhibitors of basal autophagy in HepG2 cells. This contention is supported by the ultrastructural analysis in which no autophagosomes and autolysosomes were observed in HepG2-treated cells. It is evident that the impairment in basal autophagy could lead to an accumulation of long-lived proteins and damaged organelles. In addition to its conventional role in cell survival, autophagy can also be a death promoter^42^. We have previously demonstrated that autophagy in HepG2 cells has a pro-survival effect and that autophagy inhibition inhibits cell proliferation^20^. Hence, the inhibition of the basal autophagy caused by EB-3P and EB-3D might contribute to antiproliferative action of these compounds.

In summary, EB-3D and EB-3P have multifactorial effects: 1) the inhibition of ChoKα enzyme activity, 2) downregulation of ChoKα expression, 3) AMPK activation, 4) apoptosis dependent on ER stress, 5) inhibition of basal autophagy, and 6) deregulation of lipid metabolism. The disruption of phospholipid homeostasis is associated with an alteration of cholesterol metabolism and cell survival. All these results indicate that EB-3D and EB-3P have a potential anti-cancer activity through the inhibition of lipid metabolism.

## Methods

### Materials

FBS and MEM were obtained from Biowest (Nuaillé, France). The TLC plates, protease-inhibitor cocktail, fluorimetric intracellular ROS kit, mitochondrial membrane potential kit, phosphatidylcholine assay kit, and caspase 3 assay kit were from Sigma-Aldrich (Madrid, Spain). The Muse Annexin V & Dead Cell Assay was from Merck Chemicals & Life Science (Madrid, Spain). [Methyl-^14^C]choline, [1,2-^14^C]acetic acid and [2-^3^H]glycerol were from Perkin Elmer (Madrid, Spain). Mini-PROTEAN® TGX Stain-Free™ Protein Gels, Trans-Blot® Turbo™ Mini PVDF and Clarity™ Western ECL substrate were from Bio-Rad Laboratories, Inc. (Madrid, Spain). Monoclonal anti-human primary antibody ChoKα and polyclonal β-actin were from Santa Cruz Biotechnology, Inc. (Heidelberg, Germany). Monoclonal anti-human primary antibodies (p62, AMPKα, Phospho-AMPKα, CHOP, IRE1α), polyclonal anti-human primary antibodies (LC3A/B and Beclin-1) and horseradish peroxidase (HRP)-linked secondary IgGs were from Cell Signaling Technology (Danvers, MA, USA). Polyclonal anti-human antibody, SREBP-2, and monoclonal anti-human antibodies LDLR and HMGCR were from Abcam (Cambridge, MA, USA).

### Cell culture

The human hepatoma HepG2 cell line was obtained from the European Collection of Animal Cell Cultures (Salisbury, UK). The cells were cultured in MEM containing 10% heat-inactivated FBS supplemented with 2 mM L-glutamine, 1% non-essential amino acids, 100 U·mL^−1^ penicillin and 100 μg·mL^−1^ streptomycin, in a humid atmosphere with 5% CO_2_ at 37 °C, and subcultured at a ratio of 1:10 once a week.

### Cell proliferation assay

HepG2 cells were seeded onto 96-well plates (10,000 cells/well) and maintained in MEM containing 10% FBS for 24 h. Then, the culture medium was replaced with fresh medium/10% FBS and the cells were incubated up to 48 h in the absence or presence of different amounts of ChoKα inhibitors before analyses. The antiproliferative effect of the distinct compounds was assessed by the crystal violet-staining assay using a cell-number-based standard curve as previously reported^43^. The absorbance of crystal violet in each well was measured at a wavelength of 590 nm directly in plates using a microplate reader (HTX Microplate Reader BioTek Instruments, Vermont, USA).

### Choline uptake assay

Choline uptake was determined as previously reported^44^. Briefly, HepG2 cells were incubated for 10 min or 24 h at 37 °C either in a medium containing different concentrations of ChoKα inhibitors or with no supplement as controls. The medium was then removed and the cells immediately exposed to a medium containing [methyl-^14^C]choline (16 μM, 31 Ci·mol^−1^) for 5 min at 37 °C. The incorporation of choline was stopped by medium aspiration followed by two washes with ice-cold PBS containing 580 µM choline. The cells were solubilized in NaOH 0.1 N and an aliquot used to determine the total amount of radiolabel taken up by the cells.

### Metabolic labelling assays

Cells were incubated both in the presence and absence of different amounts of ChoKα inhibitors for 24 h. Either [methyl-^14^C]choline (60 μM, 27 Ci·mol^−1^), [1,2-^14^C]acetic acid (35 μM, 29 Ci·mol^−1^) or [2-^3^H]glycerol (25 μM, 80 Ci·mol^−1^) were added during the last 4 h of the incubation period. Lipid biosynthetic activity was estimated according to the level of incorporation of the radiolabel of each exogenous precursor into the corresponding lipid. Lipids were extracted from the cells following the procedure of Bligh and Dyer^45^. The different phospholipids were separated on silica-gel 60 G TLC plates using a mixture of chloroform/methanol/acetic acid/water (60:50:1:4, v/v) as a solvent, and neutral lipids were separated by TLC using a solvent of n-hexane/ethyl ether/acetic acid (80:20:2, v/v). The lipid spots were made visible by exposure to iodine vapour, and the scraped spots were radiometrically measured by liquid scintillation using a Beckman 6000-TA counter (Madrid, Spain).

### Immunoblotting analysis

Cells growing in the log phase were incubated for 24 h with MEM/10% FBS in the absence or presence of 30 μM of the assayed compounds. The cells were washed twice, scraped into ice-cold PBS (pH 7.4) and centrifuged at 100 × g for 10 min at 4 °C. The cell pellets were suspended in 0.1 mL ice-cold lysis buffer consisting of 50 mM Tris-HCl (pH 7.4), 150 mM NaCl, 1% Triton X-100 and a protease inhibitor cocktail, and incubated on ice for 30 min with occasional shaking. Cell lysates were centrifuged at 10,000 × g for 15 min at 4 °C and supernatants were stored at −80 °C until used; an aliquot was taken to determine the protein concentration. Equal amounts of lysate protein were separated by SDS-PAGE and transferred onto PVDF membranes. Pre-stained protein molecular-weight markers were used during electrophoresis. Membranes were blocked in TBS containing 5% non-fat dried milk and 0.05% Tween-20 for 1 h, and then probed with anti-human primary Igs (1:1,000) in 3% BSA-blocking buffer at 4 °C overnight. After several washes in TBS containing 0.05% Tween-20, the membranes were incubated with the corresponding HRP-conjugated IgG (1:2,000) as a secondary antibody for 1 h. Immunoreactive proteins were detected using ECL substrate and the membranes were imaged using the Molecular Imager ChemiDocTM MP System (Bio-Rad Laboratories, Inc., Madrid, Spain). Following incubation with an antibody-stripping solution consisting of 60 mM Tris-HCl (pH 6.8), 100 mM β-mercaptoethanol and 2% SDS for 30 min at 60 °C, the blots were probed with polyclonal anti-human β-actin (1:1,000) to monitor the loading and transfer of the blotted samples. Densitometric analyses were carried out using ChemiDocTM MP System software.

### Mitochondrial membrane potential assay

The MMP was determined by using the fluorescent probe JC-10 dye following the manufacturer’s recommendations. Briefly, cultured cells were exposed up to 30 µM ChoKα inhibitors for 24 h or to 1 μM FCCP for 2 h as a positive control. After this incubation period, JC-10 dye solution was added to each sample and the fluorescence intensity (λ_ex_ = 490/λ_em_ = 525 nm) and (λ_ex_ = 540/λ_em_ = 590 nm) was monitored. Mitochondrial depolarization is indicated by a decrease in the red/green fluorescence intensity ratio.

### Apoptosis assay

The induction of apoptosis was determined by flow cytometry using the Annexin V and Dead Cell kit. HepG2 cells were incubated for 24 h either in a medium containing 30 µM ChoKα inhibitors or with no supplement as controls. Staurosporine (0.5 µg·mL^−1^) was used as a positive control. This kit is based on the detection of PS, on the surface of apoptotic cells, by a reagent containing fluorescently labelled Annexin V and 7-amino-actinomycin (7-AAD). This kit can distinguish between four cell populations: viable cells (Annexin V^−^ and 7-AAD^−^), early apoptotic cells (Annexin V^+^ and 7-AAD^−^), late apoptosis or dead cells (Annexin V^+^ and 7-AAD^+^) and necrotic cells (Annexin V^−^ and 7-AAD^+^).

### Transmission electron microscopy

HepG2 cells were plated in six-well dishes and allowed to grow for 24 h. A vehicle (control) or 10 and 30 µM EB-3D or EB-3P was added for 24 h. Cells were collected using trypsin and centrifuged at 1,500 r.p.m. for 5 min in MEM/10% FBS. Cell pellets were fixed in 2.5% glutaraldehyde +2% paraformaldehyde in 0.05 M cacodylate buffer for 4 h at 4 °C. The samples were washed three times with cacodylate buffer and postfixed in an aqueous solution of 1% OsO_4_ containing 1% potassium ferrocyanide for 1 h at 4 °C in darkness. The following washes were done: 0.15% tannic acid in cacodylate buffer, cacodylate buffer, and H_2_O, all at room temperature. The samples were left in 2% uranyl acetate for 2 h and washed several times with H_2_O. Dehydration in ethanol solutions rising from 50% to 100% was done at 4 °C. The samples were embedded in resin (EMbed 812/100% ethanol (1/1)) for 60 min at room temperature, the same resin at a 2/1 ratio for 60 min, and then resin without ethanol overnight. For polymerization, the samples were incubated in pure resin for 48 h at 60 °C. Ultrafine sections (50–70 nm) were cut using a Leica Ultramicrotome R and contrasted using 1% aqueous uranyl acetate for 5 min and lead citrate in a CO_2_-depleted atmosphere for 4 min^46^. A Zeiss Libra Plus 120 electron microscope was used to visualise the sections.

### Other analyses

The intracellular ROS were determined by using the fluorimetric intracellular ROS kit following the manufacturer’s recommendations. This kit provides a fluorogenic sensor which react with ROS, resulting in a fluorometric product proportional to the amount of ROS present in live cells. The assay was performed in 96 multiwell plates, with detection at λex = 650/λem = 675 nm.

The levels of PC were quantified following the manufacturer’s instructions for the PC levels kit after treatment of cells with or without inhibitors.

The caspase 3 fluorimetric assay kit was used to measure caspase 3 activity in cell extracts. The peptide substrate of this kit, acetyl-Asp-Glu-Val-Asp-7-amido-4-methylcoumarin, is hydrolyzed in the presence of caspase 3 resulting in the release of the fluorescent 7-amino-4-methylcoumarin moiety, which is detected at λex = 360/λem = 460 nm.

ATPlite assay kit from PerkinElmer was used to determine the intracellular levels of ATP. This kit is based on the generation of luminescence initiated by the reaction of ATP with luciferase and D-luciferin.

Protein concentrations were determined by the Bradford’s method^47^ using BSA as standard.

### Statistics

The results are expressed as means ± SEM. A one-way ANOVA was conducted with *post hoc* comparisons by Scheffé’s test (SPSS 13.0). P < 0.05 is considered statistically significant.
